# *Oncopeltus*-like gene expression patterns in *Murgantia histrionica*, a new hemipteran model system, suggest ancient regulatory network divergence

**DOI:** 10.1186/s13227-020-00154-x

**Published:** 2020-04-22

**Authors:** Jessica Hernandez, Leslie Pick, Katie Reding

**Affiliations:** Department of Entomology, University of Maryland, 4291 Fieldhouse Drive, College Park, MD 20742 USA

**Keywords:** Harlequin bug, Hemiptera, Insect model system, Segmentation, Pair-rule genes, E75A, *Murgantia histrionica*, Pentatomidae

## Abstract

**Background:**

Much has been learned about basic biology from studies of insect model systems. The pre-eminent insect model system, *Drosophila melanogaster*, is a holometabolous insect with a derived mode of segment formation. While additional insect models have been pioneered in recent years, most of these fall within holometabolous lineages. In contrast, hemimetabolous insects have garnered less attention, although they include agricultural pests, vectors of human disease, and present numerous evolutionary novelties in form and function. The milkweed bug, *Oncopeltus fasciatus* (order: Hemiptera)—close outgroup to holometabolous insects—is an emerging model system. However, comparative studies within this order are limited as many phytophagous hemipterans are difficult to stably maintain in the lab due to their reliance on fresh plants, deposition of eggs within plant material, and long development time from embryo to adult.

**Results:**

Here we present the harlequin bug, *Murgantia histrionica*, as a new hemipteran model species. *Murgantia*—a member of the stink bug family Pentatomidae which shares a common ancestor with *Oncopeltus* ~ 200 mya—is easy to rear in the lab, produces a large number of eggs, and is amenable to molecular genetic techniques. We use *Murgantia* to ask whether Pair-Rule Genes (PRGs) are deployed in ways similar to holometabolous insects or to *Oncopeltus*. Specifically, PRGs *even*-*skipped, odd*-*skipped, paired* and *sloppy*-*paired* are initially expressed in PR-stripes in *Drosophila* and a number of holometabolous insects but in segmental-stripes in *Oncopeltus*. We found that these genes are likewise expressed in segmental-stripes in *Murgantia,* while *runt* displays partial PR-character in both species. Also like *Oncopeltus*, *E75A* is expressed in a clear PR-pattern in blastoderm- and germband-stage *Murgantia* embryos, although it plays no role in segmentation in *Drosophila*. Thus, genes diagnostic of the split between holometabolous insects and *Oncopeltus* are expressed in an *Oncopeltus*-like fashion during *Murgantia* development.

**Conclusions:**

The similarity in gene expression between *Murgantia* and *Oncopeltus* suggests that *Oncopeltus* is not a sole outlier species in failing to utilize orthologs of *Drosophila* PRGs for PR-patterning. Rather, strategies deployed for PR-patterning, including the use of *E75A* in the PRG-network, are likely conserved within Hemiptera, and possibly more broadly among hemimetabolous insects.

## Introduction

Much of our understanding of the genetic and molecular mechanisms underlying animal development, morphology and physiology is based on studies of a handful of well-established model systems. Approaches in comparative biology have built upon discoveries from these model systems, revealing highly conserved genes and pathways shared across phyla. For example, based on work in the pre-eminent model organism *Drosophila melanogaster,* this approach revealed unexpected and conserved roles of *Hox* genes in embryonic development and *Pax* genes in eye development throughout Metazoa (reviewed in [1–5]). However, to discover novel genes and mechanisms, new model systems are required [6, 7]. Insects are ideal model systems not only because of their short life cycles and embryos that can be collected in large numbers and readily manipulated, but also because insects represent the majority of terrestrial species on our planet, occupy diverse habitats on land and water, and display extensive diversity in morphology, behavior and more [8]. Most emerging insect model systems have focused on the group of holometabolous insects, to which *Drosophila* belongs (for examples, see [9–13]).

Hemiptera, a close outgroup to holometabolous insects, is a large order of insects, with > 85,000 species identified to date [14]. The piercing-sucking feeding mechanism seen within this clade enables many species to feed on plants, making many of them agricultural pests (e.g., aphids, scale insects, white flies, kudzu bug, stink bugs, psyllids), and even on humans in the case of kissing bugs, allowing them to vector human disease. The milkweed bug, *Oncopeltus fasciatus*, is easily reared in the lab on a simple diet of sunflower seeds and water, and has recently emerged as an excellent model system for this group of hemimetabolous insects. It was noted as early as the 1970s that “*[t]here are very few basic biological or entomological problems for which the milkweed bug would not be a good experimental animal*” [15]. In fact, prior to the explosion of *Drosophila* molecular genetics, *Oncopeltus* was used as a model for developmental studies [16]. More recently, the development of tools to examine and manipulate gene expression [17–19] and publication of transcriptomes and genome sequences [20–22] has moved *Oncopeltus* to “prime model status” [23]. Because of the practical importance of Hemiptera, the diversity within this order, its phylogenetic position, and because progress on *Oncopeltus* has revealed novelties in morphology and molecular mechanisms (see below), we are seeking to establish additional models within this clade and report here the development of the harlequin bug, *Murgantia histrionica,* as a new hemipteran model species. Unlike *Oncopeltus*, *Murgantia* is a major pest of cruciferous vegetables and thus the development of genetic tools for this species may be useful for pest control.

Research on early embryonic body plan establishment in *Drosophila* has revealed that sets of regulatory genes act in a hierarchy to sequentially subdivide the embryo into increasingly specified body regions, culminating in the establishment of repeated segmental units along the anterior–posterior axis [24]. Mutations in the pair-rule genes (PRGs) result in loss of alternate body segments and most PRGs are expressed in stripes in the primordia of every other segment (PR-stripes)—the alternate segmental units missing in corresponding mutants (e.g., [25–27]. Segment polarity genes, the next set of genes to act in the regulatory hierarchy, impact the development of equivalent portions of each body segment and are expressed in stripes in the primordia of every segment (segmentally). PRGs regulate the expression of segment polarity genes such as *engrailed* and *wingless*, with alternate segmental stripes missing in PRG mutants [28, 29]; for example, in *Drosophila*, Fushi tarazu (Ftz) directly regulates the expression of alternate *engrailed* stripes [30, 31].

*Drosophila* is a holometabolous insect with a derived mode of segmentation in which all segments are specified more or less simultaneously during the blastoderm stage. However, in most insect orders, segments are added sequentially to the germband, with only the most anterior segments specified at the blastoderm stage (sequential segmentation, reviewed in [18, 32, 33]). Given this major change in the process of segment formation between *Drosophila* and more basally branching insects, it seemed likely that the genetic mechanisms underlying segment formation would likewise differ. However, the finding that orthologs of many PRGs are expressed and function as typical PRGs—specifying alternate segmental units—in the sequentially specifying beetle *Tribolium castaneum*, demonstrated that PR-patterning per se is conserved in sequentially segmenting species. Further, many of the genes involved in this process play similar roles despite differences in the morphological steps involved in segment addition [34]. In particular, in beetles, orthologs of two PRGs (*paired* (*prd)* and *sloppy*-*paired* (*slp*)) function in the same way as their *Drosophila* orthologs, with knockdown leading to ‘classical’ PR-defects in which alternate segments are missing [34–36]. Other PRG orthologs have dual roles in sequential segmentation: three (*even*-*skipped (eve), runt (run),* and *odd*-*skipped* (*odd*)), which are expressed and function exclusively in PR-stripes in *Drosophila*, are expressed in the segment addition zone (SAZ) in sequentially segmenting holometabolous insects in addition to being expressed in PR-stripes in these species [34, 37]. Strong knockdown of these genes results in truncated embryos, with extreme cases of ‘head-only’ embryos resulting after knockdown of *eve* in beetles, while weaker knockdown, in which the germband was able to elongate, revealed additional ‘classical’ PR-like roles for these genes [34, 37]. Additionally, expression data from several other holometabolous insects indicate conserved roles for several orthologs of *Drosophila* PRGs in pair-rule patterning. For example, *eve, run,* and *prd* in the honey bee *Apis mellifera; eve, odd,* and *run* in the silk worm *Bombyx mori* and the parasitoid wasp *Nasonia vitripennis*, were also shown to have PR-expression patterns [10, 38–42]. The expression and/or function of other PRGs (*ftz*, *hairy*, *odd*-*paired* and *ftz*-*f1*) varies even within holometabolous insects [34, 37, 43, 44]. In sum, within the Holometabola, orthologs of five PRGs—*eve*, *odd*, *prd*, *slp*, and *run—*are expressed and function in PR-stripes in the primordia of alternate segmental units in broadly divergent species within this clade.

**Fig. 1:**
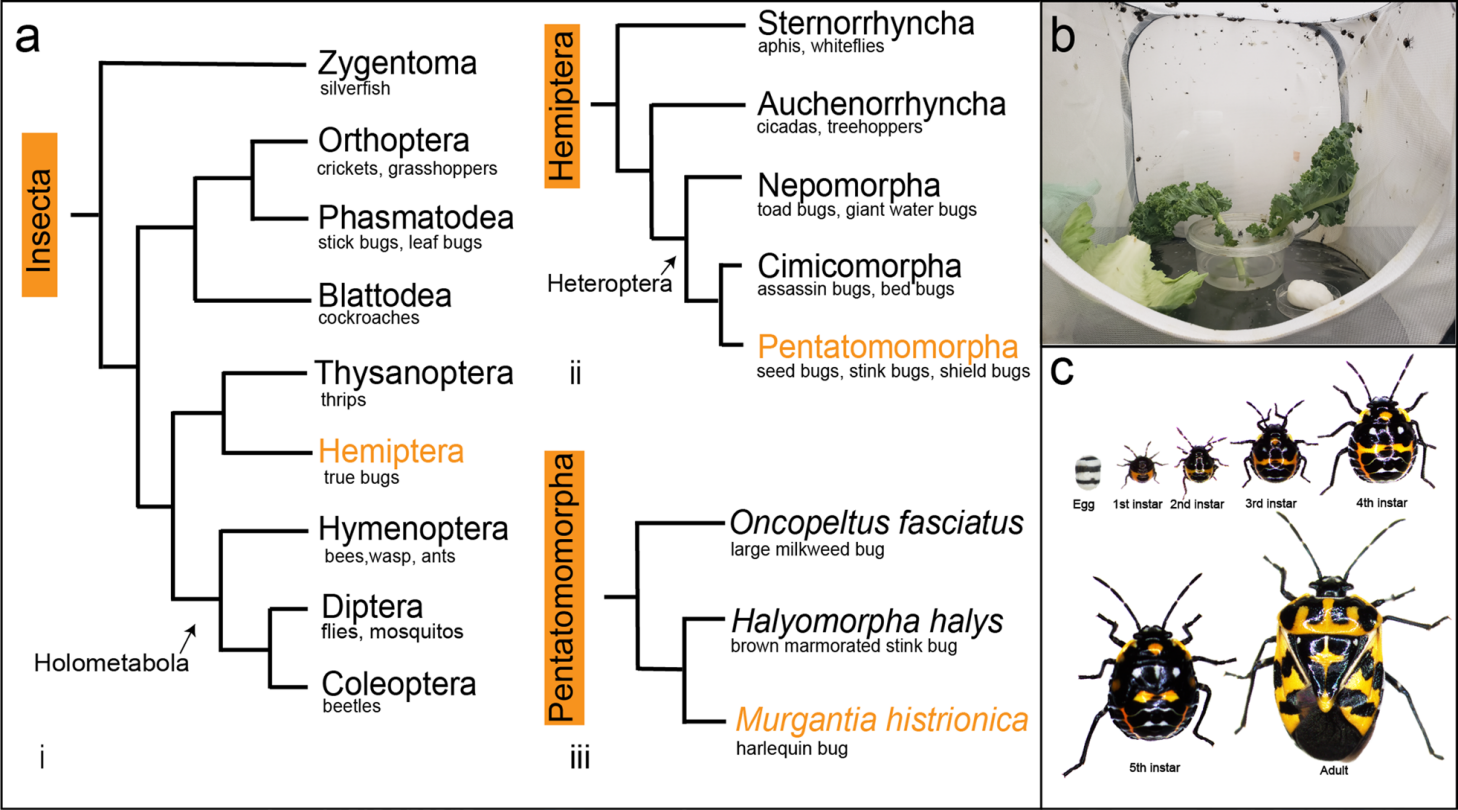
Phylogenetic context and life cycle of *Murgantia histrionica*. **a** Phylogenetic context of *Murgantia*. Trees are based on Misof et al. [54] and Li et al. [51]. (i) A condensed cladogram that includes some insect orders. *Murgantia* belongs to the order Hemiptera (colored orange) (ii) Shows a cladogram of Hemiptera, including some major suborders: Sternorrhyncha, Auchenorrhyncha and Heteroptera. *Murgantia* is a member of Heteroptera and the Pentatomomorpha superfamily (orange text). (iii) *Murgantia’s* relationship to *Oncopeltus fasciatus* (large milkweed bug) and *Halyomorpha halys* (brown marmorated stink bug). **b** Laboratory cage set up for *Murgantia*. Embryos are allowed to hatch and grow to the second instar in a small petri dish with a piece of wet cotton and kale. They are provided kale three times a week and kept in mesh cages (12 × 12 × 12 in) at 25 °C. A source of water can be provided (e.g. a wet cotton). **c** The life cycle of *Murgantia*. Our lab colony was observed to have five nymphal stages

In contrast to this, recent studies in the large milkweed bug, *Oncopeltus fasciatus* (Hemiptera: Lygaeidae), revealed that orthologs of *Drosophila* PRGs are expressed segmentally, rather than in a PR-like manner, more similar to segment polarity genes in *Drosophila* [45, 46]. Yet PR-like patterning does occur in this species; *Of*-*E75A* is expressed in PR-stripes and knockdown resulted in PR-like defects [47]. Interestingly, *E75A* does not have PR-function in *Drosophila* [48, 49]. Thus, PR-patterning appears to involve a different set of regulatory genes in *Oncopeltus* than in holometabolous insects. To ask whether these differences in the expression and function of PRG-orthologs in *Oncopeltus* compared to holometabolous insects is species-specific or a feature of other hemipterans, we used the harlequin bug, *Murgantia histrionica*, as a model system. With an estimated divergence time of ~ 200 million years from *Oncopeltus* [50–52], *Murgantia* and *Oncopeltus* are distant relatives both belonging to the order Hemiptera and infraorder Pentatomomorpha (Fig. 1a), a species-rich group with over 40,000 members [53, 54]. Here we show that *Murgantia* can be adapted to laboratory conditions and maintained as a breeding colony for many generations. We focused on a set of genes that are diagnostic of differences between holometabolous insects and *Oncopeltus* (*E75A, eve, odd, runt, prd,* and *slp*) and found that *Murgantia* orthologs are expressed in an *Oncopeltus*-like fashion. This suggests that the roles of these genes are shared among Hemiptera and possibly other hemimetabolous insects. In addition to its utility for basic research, *Murgantia* is a pest of cruciferous vegetables and progress on molecular genetics approaches in this species can lead to novel pest control strategies.

## Results

### Establishment of *Murgantia* as a lab model system

To serve as a viable model system for molecular genetic studies, organisms must be amenable to long term lab culturing, have a reasonably short life cycle, produce a large number of embryos, and molecular protocols must work well [6]. Before proceeding with genetic analyses, we assessed the feasibility of rearing *Murgantia* in the lab. We established lab colonies from field-collected *Murgantia* in summers of 2016 and 2017 and have maintained that colony continuously since that time. As described in the Methods, *Murgantia* were reared on a diet of fresh organic kale and cabbage leaves with wet cotton as a water source at 25 °C, 50% relative humidity, 16:8 light: dark cycle (Fig. 1b). Males can be readily distinguished from females by the lateral lobes of the genital capsule which are externally visible (arrows, Additional file 1: Fig. S1). Adults typically began mating 10–15 days after their last molt. In each cage, adults began laying eggs soon thereafter, first 1–2 clutches (12–24 eggs) per cage in 24 h and up to 7–8 clutches (84–96 eggs) in 24 h during their egg-laying peak, decreasing after 3 weeks. Embryos usually hatched 5–6 days after egg laying (AEL). A total of five nymphal stages were observed (Fig. 1c). The first instar lasted 4–5 days; the second, third and fourth instars each lasted 6–7 days; and the fifth and final nymphal instar lasted about 11–13 days. Thus, under our rearing conditions, development from egg to adult took ~ 38–45 days. While rearing on fresh food was effective, we have also tested whether *Murgantia* can be grown on a diet of seeds, similar to *Oncopeltus*. Preliminary results show that adults can survive on water and seeds (spider flower plus sunflower) though fecundity appears to be lower than those reared on kale/collard greens and cabbage (data not shown).

**Fig. 2:**
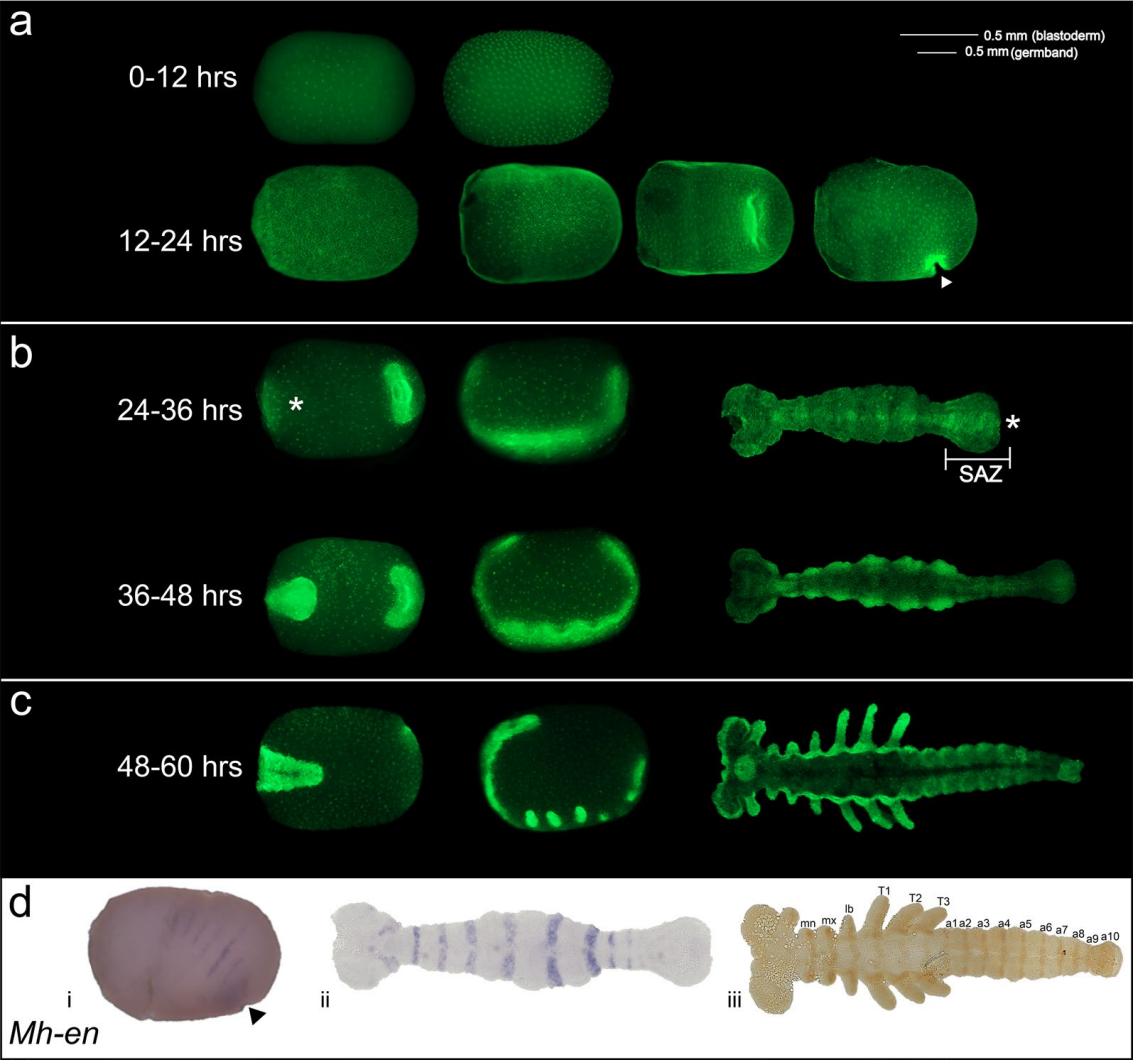
Embryogenesis and *engrailed* expression of *Murgantia histrionica.* Photos of embryos representing the first 60 h of *Murgantia* embryogenesis at 25 °C are shown. Embryos are shown with the anterior to the left. **a** During the first 12 h nuclei divide to give rise to an early, likely syncytial blastoderm stage. This continues until invagination occurs at the posterior (indicated by arrow) and the germband begins to grow along the ventral side of the embryo. **b** Germband elongation continues for the next 24 h with segments added at the segment addition zone (SAZ). **c** During the next 12 h a fully elongated germband develops and appendages are visible. Asterisks indicate posterior end of germband. **d** Expression of *engrailed* in *Murgantia*. (i) *Mh*-*en* is expressed in six stripes in the late blastoderm. (ii) *Mh*-*e*n is expressed in every mature segment during germband elongation. (iii) Expression of Engrailed was examined by immunohistochemistry using the monoclonal antibody 4D9 and a fully elongated germband. (Note that this antibody can detect both En and Invected but we have not as yet isolated *Mh*-*invected* and do not know if its expression overlaps with *Mh*-*en.*)

In order to understand embryogenesis in *Murgantia,* we performed nuclear staining of embryos carefully staged at 25 °C (Fig. 2). During the first 12 h AEL, nuclear division occurs and nuclei were observed at the periphery of the yolk. At the end of the first 24 h, invagination of the germband began at the posterior with cells aggregating at this site (Fig. 2a, arrowhead). For the next 24 h, the germband elongated along the ventral side of the embryo with segments added at the posterior end in the segment addition zone (SAZ) (Fig. 2b, asterisks). In early germbands, clear morphological segmentation was observed only in the thoracic region; as the germband elongated, abdominal segments were added at the posterior, later becoming clearly morphologically segmented. At 48–60 h AEL, a fully elongated germband with gnathal and thoracic appendages was observed (Fig. 2c).

To establish whole mount in situ hybridization and immunohistochemistry in *Murgantia,* we used *engrailed (en),* which is expressed in a highly conserved pattern at the border of each segment in developing arthropods and serves as a segmental marker across species [55–58]. The major challenge to isolating and fixing embryos for visualization of gene expression was that embryos are well-protected by a thick chorion that was difficult to penetrate. We found that immersion of embryos in hot water, followed by boiling and an extended time on ice facilitated physical removal of the pseudoperculum (anterior cap of the chorion, Additional file 2: Fig. S2) which was necessary to allow fixative to access the embryo. In addition, incubation with xylene softened the chorion and facilitated dissection (see Methods for details). In late blastoderm-stage embryos, *Mh*-*en* was expressed in six stripes, suggesting that six segments are established in this species before germband elongation (Fig. 2di). *Mh*-*en* stripes were added as the germband elongated, presumably marking each segment (Fig. 2dii). Similarly, *Mh*-En was detected in segmental stripes by immunohistochemistry using monoclonal anti-Engrailed antibody 4D9, (Fig. 2diii). Overall, these experiments demonstrate that lab rearing and basic molecular genetic approaches to examine gene expression are effective in *Murgantia*. While no *Murgantia histrionica* genome has been sequenced yet, the USDA ARS has listed this species as a likely candidate for genome sequencing as part of its Ag100Pest initiative [59].

### *Murgantia E75A* is expressed in PR-stripes

**Fig. 3:**
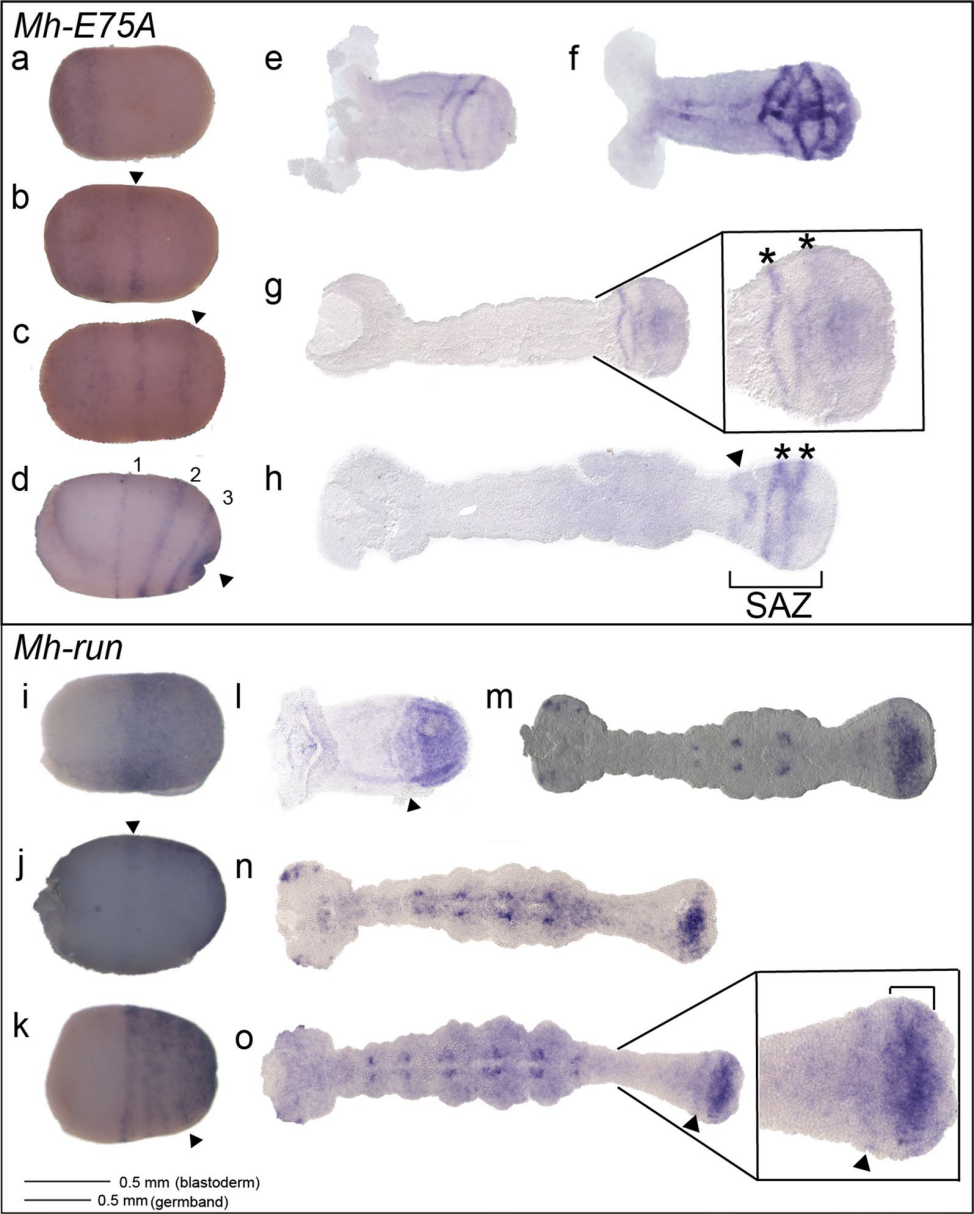
*Mh*-*E75A* and *Mh*-*run* expression. **a***Mh*-*E75A* is expressed broadly in the anterior in an early blastoderm-stage embryo (16–24 h AEL); **b***Mh*-*E75A* continues to be expressed broadly in the anterior with one stripe appearing in the middle of the embryo (arrow head); **c***Mh*-*E75A* resolves into two stripes (arrowhead points to new stripe) with continued expression at the anterior part of the embryo; **d** In late blastoderm-stage embryos (24–36 h AEL), invagination of the embryo is beginning to occur (indicated with an arrowhead). At this point *Mh*-*E75A* has resolved into three stripes. **e**–**h***Mh*-*E75A* expression during germband elongation. *Mh*-*E75A* is expressed at the segment addition zone. Asterisks highlight stripes; arrowhead indicates fading stripe. **i***Mh*-*run* is expressed in the posterior half of an early blastoderm-stage embryo. **j***Mh*-*run* resolves into one stripe with continued expression at the posterior. **k** Three stripes of *Mh*-*run* are observed in later blastoderm-stage embryos, with one stripe appearing to split. **l***Mh*-*run* was observed anterior to broad SAZ expression in some early germband-stage embryos (arrowhead); **m** In an elongating germband, *Mh*-*run* continues to be expressed in the SAZ, and was also observed in dots of expression in the thoracic segments and the head lobes; **n** In later germbands, *Mh*-*run* was still observed broadly in the SAZ; **o** Occasionally stripes (arrowhead) of *Mh*-*run* were observed anterior to SAZ expression. Embryos are shown with the anterior to the left

We chose a set of genes that are diagnostic of the difference between PRG-ortholog expression in *Oncopeltus* and in holometabolous insects to determine whether the genetic basis of segmentation in *Murgantia* is similar to one of these. *E75A* is expressed in PR-stripes in *Oncopeltus* but not in *Drosophila* and, to date, is the only PRG identified in *Oncopeltus* [46, 47]. In *Murgantia*, *Mh*-*E75A* was first detected broadly in the anterior portion of early blastoderm-stage embryos (Fig. 3a). One clear *Mh*-*E75A* stripe appeared at the center of the embryo (Fig. 3b, arrowhead), as another stripe began to resolve from the anterior domain of expression (Fig. 3b). Another stripe appeared posterior to this center stripe (Fig. 3c, arrowhead). As germband invagination began, *Mh*-*E75A* was observed in three clear stripes (Fig. 3d). Based on the finding that *en* was expressed in six stripes at this stage (Fig. 2di), we conclude that these domains of *E75A* expression correspond to the primordia of alternate segments and thus the pattern represents classic PR-expression. *Mh*-*E75A* continued to be expressed during germband elongation. In early germbands, *Mh*-*E75A* was expressed in two stripes anterior of the SAZ (Fig. 3e, f). In later germbands, one obvious stripe was observed with a second stripe resolving in the center of the SAZ (Fig. 3g, asterisks). Later, *Mh*-*E75A* continued to be expressed in the SAZ with older stripes disappearing anteriorly while two stripes remained in the center of the SAZ (Fig. 3h). The number and spacing of *Mh*-*E75A* stripes in the blastoderm, as it compares to *Mh*-*en* expression at the same stage, suggests a role in PR-patterning. The two-stripe cadence of *E75A* expression during germband elongation suggests that it has the potential to act as a PR regulator in abdominal segment patterning as well. In sum, *Mh*-*E75A* is expressed in a PR-like pattern, similar to that seen in *Oncopeltus*.

### *Mh*-*run* is expressed in stripes in the blastoderm and broadly in the SAZ

In *Oncopeltus*, *run* was the only *Drosophila* PRG ortholog that showed any PR-like expression [46], making this a particularly interesting gene to examine in another hemipteran. *Mh*-*run* was first detected broadly in the posterior half of the embryo (Fig. 3i). Expression then resolved into a broad stripe near the center of the embryo with continued expression at the posterior (Fig. 3j, arrowhead). As gastrulation began, three stripes were observed with continued expression in a posterior domain (Fig. 3k, arrowhead). This expression pattern differs from that seen in blastoderm-stage *Oncopeltus* embryos, where two early broad stripes each appear to split [46]. While we never observed two earlier such stripes of *Mh*-*run,* we cannot rule out that a similar earlier expression pattern exists. It is important to note that *Of*-*run* expression was reported to be highly dynamic; given that *Murgantia* lay fewer embryos than *Oncopeltus,* this reduced sampling could mask similar variability in expression. *Mh*-*run* continued to be expressed during germband elongation, where the expression pattern also differs from that reported in *Oncopeltus*. *Of*-*run* was shown to be expressed in roughly two-segment-wide stripes in the SAZ [46]. In early germbands, *Mh*-*run* was observed broadly in the segment addition zone (SAZ); occasionally, a stripe of expression anterior to the SAZ was also observed (Fig. 3l, arrowhead). In older germbands, *Mh*-*run* was expressed in two dots in each segment, possibly in the central nervous system, with continued expression of a thick band in the SAZ, and additional expression in the head lobes (Fig. 3m-o). This expression continued, with an occasional stripe seen anterior to the broad expression in the SAZ (Fig. 3o, arrowhead). In *Oncopeltus, Of*-*run* was observed in broad stripes in the anterior SAZ that appeared to split in two as segment primordia left the SAZ, suggesting the potential for PR modulation by *Of*-*run*. However, in *Murgantia,* such two-segment-wide stripes were never observed anterior to the SAZ. These *Mh*-*run* expression patterns in the blastoderm and during germband elongation are reminiscent of *Of*-*run* expression at these stages, though *Of*-*run* expression was more characteristic of classic PR patterning.

### *Mh*-*odd*, *Mh*-*eve* and *Mh*-*prd* are expressed segmentally

**Fig. 4:**
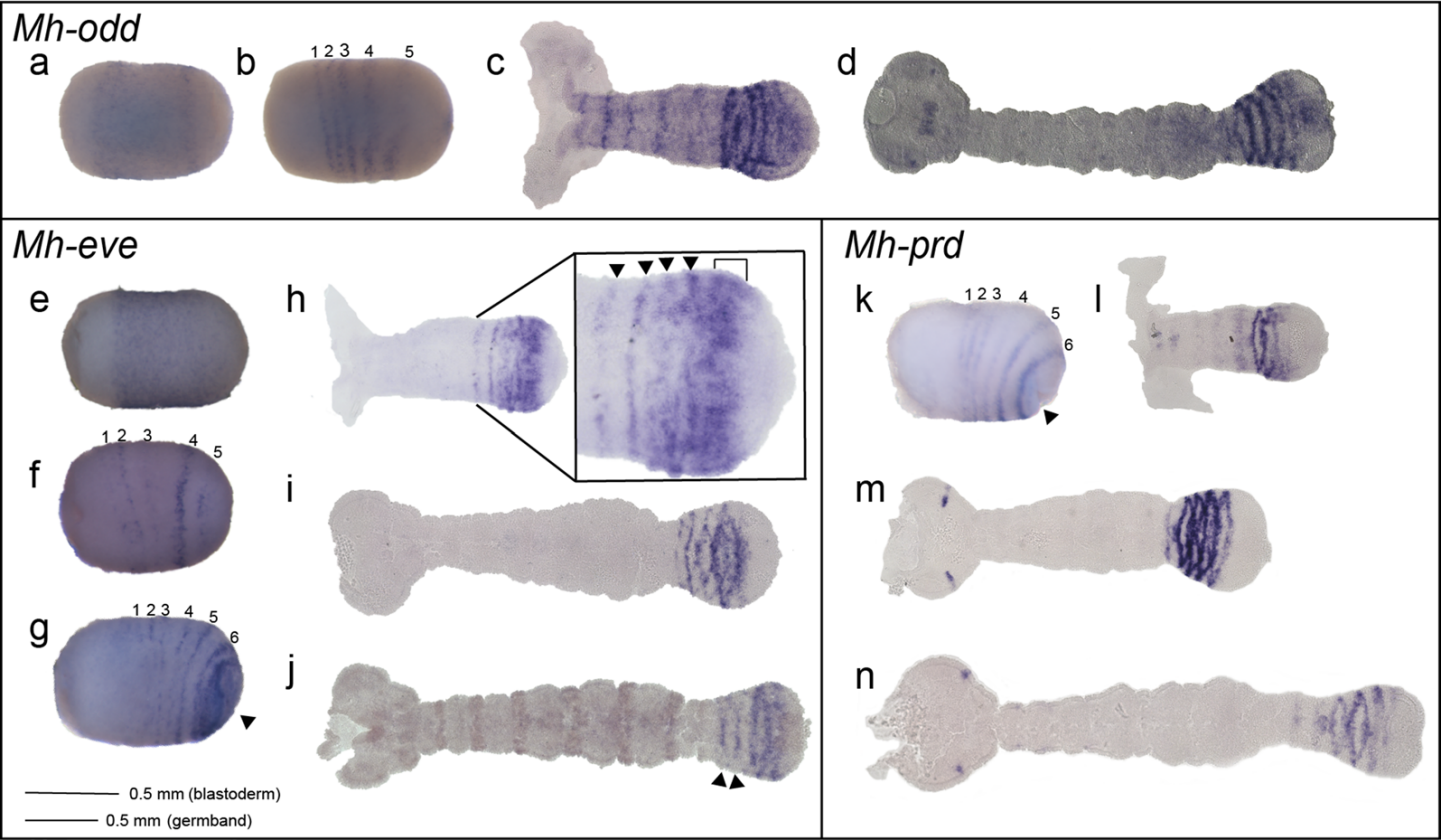
*Mh*-*odd, Mh*-*eve* and *Mh*-*prd* are expressed segmentally. **a***Mh*-*odd* was first observed in a broad region around the center of the embryo; **b** Earlier broad *Mh*-*odd* expression resolved into five stripes in a later blastoderm-stage embryo, as the germband started to invaginate at the posterior; **c***Mh*-*odd* was observed in stripes anterior to the SAZ in an early germband; **d** Anterior SAZ striped expression of *Mh*-*odd* continued in later germbands, in addition to dots of expression seen in the head lobes; **e***Mh*-*eve* was observed in the posterior two-thirds of an early blastoderm-stage embryo (16–24 h AEL). **f** A later blastoderm-stage embryo (24–36 h AEL) where *Mh*-*eve* is expressed in five stripes. **g** As invagination (indicated by arrowhead) begins to occur, *Mh*-*eve* is expressed in six stripes. **h** In an early germband, there are four stripes (arrowheads) visible with broader expression closer to the posterior SAZ (bracket). **i** In a later germband, only four distinct bands are visible and the broader domain of expression to the posterior has disappeared. **j** A double stain showing Engrailed in brown and *Mh*-*eve* in purple. Arrowheads indicate fading stripes exiting the SAZ **k***Mh*-*prd* is expressed in six stripes at germband invagination. Arrow indicates invagination. **l**–**n***Mh*-*prd* expression as germband elongation proceeds. Embryos are shown with the anterior to the left

*Mh*-*odd* was detected in a broad domain around the center of the early embryo, absent from the poles (Fig. 4a). This broad expression of *Mh*-*odd* resolved into five stripes in late blastoderm-stage embryos (Fig. 4b), in a manner similar to *Of*-*odd* [46]. *Mh*-*odd* continued to be expressed during early germband elongation and in fully elongated germbands in four stripes in the anterior SAZ with expression at the head lobes (Fig. 4c, d). This expression showed no PR-like character, similar to what was seen for *Of*-*odd.*

*Mh*-*eve* was first detected in a broad domain in early blastoderm-stage embryos, though absent from the anterior fourth of the embryo (Fig. 4e). In late blastoderm-stage embryos, *Mh*-*eve* resolved into five stripes (Fig. 4f), and six stripes were observed as gastrulation began (Fig. 4g), similar to *Mh*-*en* (Fig. 2di). In early germbands, *Mh*-*eve* was expressed anterior to the SAZ with four visible stripes and a broader region of expression in the central SAZ (Fig. 4h). In older germbands, similar, closely spaced stripes in the anterior SAZ were also observed (Fig. 4i). Double staining with anti-Engrailed antibody was performed to compare the segmental expression of *Mh*-*eve* (blue) to En (brown) during germband elongation (Fig. 4j). This revealed that En stripes appear anterior to the SAZ where *Mh*-*eve* stripes were no longer detectable (arrowheads), showing no temporal overlap of *Mh*-*eve* and *Mh*-En. In sum, *Mh*-*eve* was expressed segmentally in blastoderm and later stage embryos, with no hint of PR-register. These *Mh*-*eve* expression patterns are nearly identical to those observed for *Of*-*eve* at each stage of embryonic development examined [46, 58].

*Mh*-*prd* expression was first detected in late blastoderm-stage embryos in six stripes (Fig. 4k), presumably in the primordia of every segment. In early germband-stage embryos, *Mh*-*prd* was observed in two stripes in the anterior SAZ, as well as faintly in stripes in each more mature segment (Fig. 4l). In later germbands, *Mh*-*prd* was seen in four stripes in the anterior SAZ (Fig. 4m), very similar to the expression patterns of *Mh*-*eve* and *Mh*-*odd* at this stage (Fig. 4i, d). However, unlike *Mh*-*eve* and like *Mh*-*odd*, *Mh*-*prd* was also expressed in dots in the head lobes (Fig. 4m, n).

In sum, *Mh*-*odd,* -*eve* and -*prd* were expressed in very similar patterns to their orthologs in *Oncopeltus*. For both species, these three genes appear to be expressed in the primordia of every segment of the blastoderm, and then at a register consistent with expression in the primordia of every segment during germband elongation.

### *Mh*-*slp* is expressed in persistent segmental stripes

**Fig. 5:**
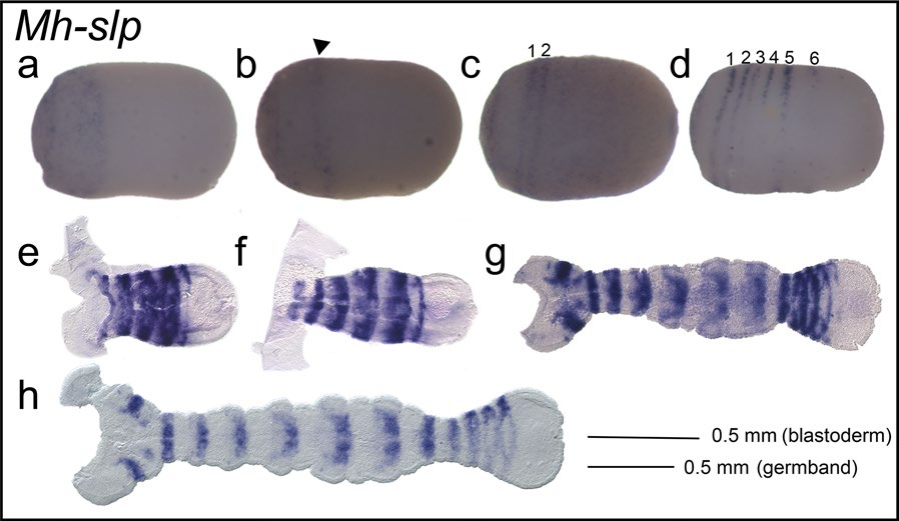
*Mh*-*slp* is expressed in persistent segmental stripes. **a***Mh*-*slp* was observed in the anterior third of an early blastoderm-stage embryo, (16–24 h AEL). **b** In an embryo slightly older than a, one stripe (arrowhead) of *Mh*-*slp* was observed. **c** Two stripes of *Mh*-*slp* were observed in an embryo older than **b**. **d** In a later blastoderm-stage embryo, six stripes of *Mh*-*slp* were observed. **e**–**h***Mh*-*slp* expression during germband elongation. *Mh*-*slp* is expressed in persistent segmental stripes during germband elongation with expression at the head lobe. Embryos are shown with the anterior to the left, ventral side down

*Mh*-*slp* was first observed in the anterior third of early blastoderm-stage embryos (Fig. 5a). This expression resolved into one stripe (Fig. 5b); later, a second stripe was observed (Fig. 5c). Stripes continued to appear posteriorly until six stripes were visible (Fig. 5d). Comparison of the anterior stripe position of *Mh*-*slp* to that of *Mh*-*odd* and *Mh*-*eve* (Fig. 4b, f) suggests that the stripes of *Mh*-*slp* seen in early blastoderm-stage embryos (Fig. 5d) are much further anterior than those of *eve* or *odd*, and thus likely correspond to pre-mandibular segments. During germband elongation, *Mh*-*slp* continued to be expressed in every segment with no expression detected in the posterior SAZ (Fig. 5e, f). In later germbands, *Mh*-*slp* was observed in every segment with expression concentrated along the midline in older segments. Additionally, expression was observed in the intercalary and antennal segments (Fig. 5g, h). This persistent segmental expression throughout the germband differs from other *Mh*-PRG orthologs and is similar to that seen for *Of*-*slp*.

## Conclusions

We have established the harlequin bug, *Murgantia histrionica*, as a versatile insect model system. *Murgantia* are easy to maintain in the laboratory on a diet of fresh organic kale/collard greens and cabbage and can be bred en masse. *Murgantia* is a hemimetabolous insect with a reasonably short life cycle (~ 6 weeks from embryo to adult) and produces enough embryos to examine gene expression. As such, this species meets all the basic requirements to serve as a model for molecular genetic analysis. Our lab previously tried to rear another pentatomid, the invasive brown marmorated stink bug *Halyomorpha halys*, and observed a collapse of our lab colony after three generations, possibly due to infection by a microsporidian [60, 61]. We therefore subjected *Murgantia* to a ‘trial period’ of lab rearing before beginning experiments to ensure that we could sustain our lab colony, and have been able to maintain a healthy lab colony for several years and at least 10 generations.

Methods for examining gene expression in *Murgantia* were adapted from *Oncopeltus* protocols and can be performed readily with these modifications on a large number of samples. In addition to their utility as a model system, *Murgantia* is a pest of cruciferous vegetables (cabbage, kale, etc.). A native of Central America, it was first reported in the United States in 1864 [62]. After its introduction in Texas, the bug spread to other states making its way to most southern states by the late 1890s. Although it is most active during summer, it has been reported that *Murgantia* can remain active during mild winters. Over the last decade, warmer winters have led to increased survival and reproduction and larger populations in subsequent seasons [63, 64]. *Murgantia* damage crops through their piercing-sucking mechanism of feeding, which leaves white blotches on leaves and can destroy crops if populations are large. Elucidation of the genes regulating *Murgantia* development can lead to non-chemical pest control methods, such as RNA interference.

Here, we used *Murgantia* as a model system to investigate conservation and variation in PR-patterning mechanisms in insects. We established methods for in situ hybridization and immunostaining; RNAi will be tested as a tool to assess gene function in the future, based on successful use of this technique in *Oncopeltus*. Previously, we and others had shown that orthologs of *Drosophila* PRGs are expressed segmentally, rather than in PR-stripes in the milkweed bug *Oncopeltus* [45, 46, 58]. Specifically, *Of*-*eve*, *prd*, *odd*, and *slp* were each found to be expressed in segmental stripes in the blastoderm with no PR-like expression observed at any stage, while *Of*-*run* showed some hint of PR-like expression [45, 46, 58]. In contrast, the *Drosophila*-like expression of PRGs in PR-stripes, in the primordia of alternate segmental units, is shared among a number of holometabolous insects, particularly for the genes *eve, prd, slp, run* and *odd* (see Introduction). These findings led us to identify another hemipteran insect for comparative studies. We found that in *Murgantia*, *Drosophila* PRG-orthologs are expressed in segmental-stripes in blastoderm-stage embryos with varying expression patterns. *Mh*-*odd, Mh*-*eve*, *Mh*-*prd* and *Mh*-*slp* were found to be expressed in five or six stripes in the late blastoderm. During germband elongation, *eve, odd,* and *prd* were expressed in a series of stripes in the anterior SAZ showing no PR-register, with additional expression in the head lobes seen for *odd* and *prd*. *Mh*-*slp* was not observed in the posterior SAZ but was found in persistent segmental-stripes during elongation, marking every mature segment. These expression patterns bear remarkable resemblance to the expression patterns of the corresponding orthologs in *Oncopeltus.* This suggests that little change in the segmentation network—with regard to any role the *Drosophila* PRG-orthologs might play in this network—occurred over the nearly 200 million years of each lineage’s independent evolution.

The similarities in expression patterns between these two species extend to the timing of segmental stripe refinement. Stahi and Chipman [65] showed that expression of four genes—including *eve* and *inv*—in segmental stripes in the *Oncopeltus* blastoderm arise nearly simultaneously, similar to blastoderm segmentation in *Drosophila*. While we cannot be confident about the precise timing of segmental stripe refinement in *Murgantia*, we did not observe many intermediate striped expression patterns, suggesting that segmental stripes of *Mh*-*en,* -*odd,* -*eve,* and -*prd* arise nearly simultaneously in the blastoderm in this species as well. This pattern is in contrast to the more extreme short-germ mode of segmentation in which segmental stripes arise in an anterior to posterior order. Future studies of blastoderm segmentation in *Murgantia* could therefore provide further insight into the evolution of different modes of segmentation.

In *Oncopeltus*, Erezyilmaz et al. [47] showed and we confirmed [46] that *E75A*, which is not a PRG in *Drosophila*, is expressed in typical PR-stripes. This nuclear receptor *E75A* does not have PR-expression in *Drosophila* and is not known to function in segmentation in any holometabolous insects. Here, we show that *Mh*-*E75A* is expressed in a PR-like pattern during development, the first indication that this gene might be involved in regulating segmentation outside of *Oncopeltus*. Specifically, *Mh*-*E75A* is expressed in three stripes in the blastoderm, presumably in the primordia of every other segment. During germband elongation *Mh*-*E75A* is expressed in two stripes anterior to the SAZ that disappear in older segments, an expression pattern reminiscent of *Of*-*E75A*. Thus, it seems that there is a different set of genes acting as PRGs within Hemiptera. Whether or not this difference indicates a distinct rift in patterning mechanisms between holometabolous groups and more basally branching insects, or is simply a feature of hemipterans, remains to be investigated.

Here we have found a high level of conservation in expression patterns of PRG-orthologs and *E75A* in two distantly related hemipterans. While it is not surprising to find greater conservation of expression patterns between these two species than between either and *Drosophila*, the degree of conservation observed helps provide needed evolutionary context which is missing when network components are examined in highly diverged species in isolation. Thus far, it appears that genes responsible for PR-patterning are wholly different in *Oncopeltus* and *Murgantia* as compared to holometabolous insects. It will be of particular interest to examine other species in the hemipteran clade to determine if a broader hemipteran segmentation network exists. Future studies of the segmentation networks operating in even more basally branching species will help determine whether an *Oncopeltus* and *Murgantia*-like network is ancestral within insects or is Hemiptera-specific. These studies will provide insight into the timing of evolutionary transitions in core body patterning regulators and the mechanisms by which such changes have occurred.

## Methods

### Laboratory cultures, life cycle and embryo collection

*Murgantia* were collected from kale, cabbage, and spider flower (*Cleome* spp.) at local gardens. In one year, a garden plot was planted only with *Cleome spinosa* (Southern Exposure Seed Exchange) which was very effective at attracting *Murgantia*, suggesting its utility as a trap crop for this species. In the lab, *Murgantia* were reared in mesh cages (12 × 12 × 12 in) at 25 °C. A total of eight cages were kept at once, four adults cages (~ 70 adults per cage) and four growing nymph cages. Each cage was given one organic kale/collard leaf and one cabbage leaf three times a week. Cages were also supplied with wet cotton as a water source and were lightly misted every other day. Embryos were collected from adult cages to set up new cages. Embryos were kept in small petri dishes, allowed to hatch and grow to the second instar in the dish and later transferred to a clean mesh cage (Fig. 1b). This was done every 2–3 months to keep the colony going. Cages were generally kept for 4 months, after which their fecundity dramatically decreased. Used cages were washed with soapy water followed by bleach and rinsed well with tap water. To collect embryos, fresh food was placed in adult cages and embryos were collected from the leaves. For gene expression analysis embryos were collected during a 8–12 h window and fixed 12–17 h after collection. As we observed no egg laying during the nighttime all collections were done during daytime. Since females lay only 12 embryos at a time a collection window of at least 8 h was necessary to collect enough embryos for experiments. To determine relative ages of embryos, the invagination pore and extent of germband elongation were compared between embryos. Later experiments revealed that Kimwipes could be used to collect embryos by pinning them to the cages with pushpins. In order to count the number of nymphal instars for this species, individual hatchlings were kept in 2 oz. cups with small holes in the lids and cup. A piece of fresh kale was given every day along with a piece of wet cotton. Cups were observed every day and each molt recorded until adulthood. A total of five nymphal stages were observed (Fig. 1c).

### cDNA, gene isolation and probe synthesis

#### RNA extraction and cDNA preparation

Embryos were collected every 24 h for RNA extraction. Approximately 40-50 *Murgantia* embryos were collected within 24 h, homogenized in 150 ul TRIzol solution (Invitrogen), and stored at − 80 °C in preparation for RNA extraction. RNA extraction was done as described by the manufacturer. A total of 1 ug RNA template was used for cDNA synthesis. cDNA was prepared following NEB’s standard protocol. Briefly, 1ug RNA template was combined with dNTPs (New England Biolabs), Random Primer Mix (New England Biolabs), and water. Tubes were incubated at 65 °C for 5 min to denature the RNA and immediately transferred to ice. Transcriptase buffer and RNAse inhibitor (New England Biolabs) were added and incubated at 37 °C for 2 min. Finally, M-MuLV Reverse Transcriptase (New England Biolabs) was added, and the reaction was incubated at 37 °C for 50 min followed by 15 min at 70 °C to inactivate the enzyme.

### Probe synthesis

DNA templates for antisense RNA probes were generated by PCR using reverse primers containing the T7 polymerase promoter sequence. Digoxigenin-labeled RNA probes were synthesized from 200 ng of purified PCR product with digoxigenin labelling mix (Roche), RNase inhibitor, and T7 polymerase. RNA transcription reactions were incubated at 37 °C for 2 h, then RNA was precipitated with 100% cold ethanol and 6 M LiCl and incubated overnight at − 20 °C. The pellet was washed with 70% ethanol, dried, and dissolved in 20 μl of nuclease-free water. In order to ensure that the pellet was completely dissolved, tubes were incubated at 37 °C for 5–10 min.

### Gene isolation

*Mh*-PRG orthologs were isolated using degenerate primers or primers matching orthologs from *Halyomorpha halys (Hhal)*, based on sequences annotated in the i5k’s publicly available draft genome [66]. Rapid Amplification of cDNA Ends (RACE) was performed when necessary. *Mh*-*en, eve, E75A,* and *prd* were isolated by degenerate PCR with Taq polymerase (see Additional file 3: Table S1 for primer sequences; degenerate primers used to isolate *Mh-eve* from [67]). 3′ and 5′ RACE were used to extend *Mh*-*en, eve, E75,* and *prd* using the FirstChoice RLM-RACE Kit (Invitrogen) or the SMARTer^®^ RACE 5′/3′ Kit (Takara Bio). *Mh*-*slp, run, and odd* were isolated with *Hhal* primers, and the SMARTer^®^ RACE 5′/3′ Kit (Takara Bio) was used to extend the 5′ and 3′ ends of *Mh*-*odd*. Orthology was determined by phylogenetic and sequence analysis using amino acid sequences of paralogs and orthologs of the target gene from various insect species (Additional file 4: Fig. S3; Additional file 5: Fig. S4). Gene trees were constructed for each ortholog using the following homologs. *en*: *en* and *invected; E75A*: *E75A* and *EcR*; *eve: eve, labial,* and *proboscipedia; runt*: *runt, lozenge, runxA,* and *runxB; odd: odd, brother of odd with entrails limited (bowl),* and *sister of odd and bowl (sob)*; *prd*: *prd, gooseberry (gsb)*, and *gooseberry*-*neuro* (*gsb*-*n*). Gene structure schematics show the regions of each gene that were isolated in this study (Additional file 6: Fig. S5). Note two isoforms of *Mh*-*prd* were found, as in *Oncopeltus*. These differ in their paired box with an insertion of 75 bp found in one isoform (Additional file 6: Fig. S5e). In some cases, the complete coding sequence was isolated as well as the 5′ or 3′ untranslated region (UTR). The sequences have been deposited to GenBank with the following accession numbers: *Mh-E75A* (MT235247), *Mh-eve* (MT235248), *Mh-slp* (MT235249), *Mh-en* (MT235250), *Mh-prd-A* (MT235251), *Mh-prd-B* (MT235252), *Mh-odd* (MT235253), and *Mh-run* (MT235254).

### Embryo fixation

*Murgantia* embryos were fixed using the protocol for *Oncopeltus* embryo fixation [46, 58, 68] with some modifications. Appropriately aged embryos were carefully separated from each other under a dissecting scope using forceps and placed in 2 ml tubes (~ 35–40 embryos/tube). To begin fixation, 600 ul of boiling water was added to each tube, and tubes were submerged in boiling water for 3 min. Tubes of embryos were then immediately submerged in ice for 15–20 min. Water was removed and replaced with PBST (0.01 M phosphate buffered saline with 0.05% TWEEN-20). Under a dissecting microscope, the pseudoperculum (or anterior “cap”) of the chorion was removed using fine-tipped forceps (Additional file 2: Fig. S2). PBST was removed and replaced with 1.2 ml of 1:1 12% paraformaldehyde (PFA):heptane. Tubes were shaken at 200 rpm for 20 min. PFA (bottom layer of liquid) was removed and replaced with 600 ul of methanol. The heptane:methanol was removed, and embryos were washed three times with 100% methanol. 1.2 mL 1:1 xylene: methanol was added to each tube and tubes were rocked for 1 h at RT to soften the chorion for manual dissection. Xylene:methanol was removed, embryos were washed three times with methanol and stored in methanol at − 20 °C.

As dissecting embryos out of their eggshells is fairly time-consuming, this was usually done several days ahead of starting an in situ hybridization. Embryos were removed from storage at − 20º C and gradually washed from 100% methanol into PBST (3 min per wash in 100%, 75%, 50%, 25% methanol diluted in PBST), followed by one 3 min wash in PBST and three rinses with PBST. The chorion was manually removed under a dissecting microscope using forceps, and embryos were then washed gradually back into 100% methanol using the aforementioned series of washes in reverse order. Embryos were washed three times with 100% methanol, methanol was removed and replaced with 1.2 ml of 1:1 methanol:heptane in order to remove the vitelline membrane by inverting and manually shaking the embryos in the tubes for 30-45 s. The methanol:heptane mixture was removed, and embryos were washed two times with 100% methanol. The embryos were washed into PBST using the same methanol gradient as above. Embryos were washed three times with PBST and fixed for 1 h in 4% PFA. After fixation, embryos were washed three times with PBST, washed gradually back into 100% methanol, and stored at − 20 °C for later use.

### In situ hybridization and SYTOX green nuclear staining

The in situ hybridization protocol described by Ben-David and Chipman [68] for *Oncopeltus* embryos was used with some modifications. Fixed embryos were gradually washed from 100% methanol into PBST (3 min per wash in 100%, 75%, 50%, 25% methanol diluted in PBST), followed by one 3 min wash in PBST and three rinses with PBST and incubated at 60º C with hybridization buffer for 3–4 h. Probes were heated for 3 min at 90 °C and placed in ice immediately. Probes were used at concentrations of 0.1–1 ng/μl. Embryos were incubated with probes at 60 °C overnight. Embryos were then incubated with pre-warmed hybridization buffer at 60 °C for 30 min, two times, followed by incubation with pre-warmed 2× SSC (saline sodium citrate) at 60 °C, 2× SSC at room temperature (RT) and finally 0.2× SSC at RT for 30 min. Embryos were washed 3 times with PBST and incubated with 10% sheep serum in PBST for 2 h at RT. Sheep serum was removed and replaced with 1:1600 dilution of anti-digoxigenin-AP Fab fragments (Roche) in 10% sheep serum and incubated for 3–4 h at RT. Embryos were washed 3 times in PBST and kept in PBST overnight at 4º C. The following day, embryos were washed with PBST for 20 min five times. In preparation for staining, embryos were washed for 15 min in alkaline phosphatase (AP) staining buffer (100 mM NaCl, 50 mM MgCl_2_, 100 mM Tris–HCl, pH 9.5 and 0.1% TWEEN-20) and 15 min in AP-PVA staining buffer (100 mM NaCl, 50 mM MgCl_2_, 100 mM Tris–HCl, pH 9.5, 0.1% TWEEN-20 and 2% polyvinyl alcohol (PVA)). BCIP and NBT (Roche) in AP-PVA was used for color detection. SYTOX green staining was done using 1:1000 SYTOX Green (Invitrogen) in PBST for 1 h at RT after fixation of embryos with 4% PFA. Blastoderm state embryos were imaged in PBST. Germbands were hand dissected, removing as much yolk as possible, and mounted in 75% glycerol in PBST.

### Immunohistochemistry

Embryos were fixed as described above, except that fixation after chorion removal was done for 30 min only. After fixation, the protocol described by Lu et al. [60] for *Halyomorpha* embryos was used except that post-fixation was not done after the staining reaction. If background staining was strong, embryos were washed in 50% methanol in PBST for 5 min and rinsed 3 times with PBST after the color reaction. The monoclonal anti-Engrailed antibody 4D9 (Developmental Studies Hybridoma Bank) was used at a 1:10 dilution. Double staining was performed by combining in situ hybridization and immunohistochemistry protocols. Protein staining was done first with some changes. First, instead of using PBST, freshly prepared PBTH (filtered 1X PBS, 0.1% Tween-20, 50 µg/ml heparin, and 250 µg/ml tRNA) was used for all steps. Additionally, RNase inhibitor was added to any incubation longer than 1 h. In situ hybridization followed antibody staining as described above. BCIP/NBT staining was done at 4 °C to decrease background staining and was monitored very closely under a dissection microscope.

### Image processing

Germbands were imaged with an AxioCam MRc camera (Zeiss) mounted on a Zeiss Axio Imager M1. Picture of germbands were taken with a 20X objective requiring image merging with Adobe Photoshop. Blastoderms were imaged in PBST using an Axiocam 506 color camera mounted on a Zeiss Discovery.V12 SteREO dissecting microscope. SYTOX-stained embryos were imaged using an Axiocam mono camera mounted on Zeiss Discovery.V12 dissecting microscope in the dark.

### Phylogenetic trees and gene structures

Protein sequences were subjected to phylogenetic analysis as described by Reding et al. [46]. Protein sequences of orthologs from various insects were collected and a multiple sequence alignment was generated using MUSCLE. Alignments were then trimmed using AliView and exported as a FASTA file to TOPALi v2.5 where a phylogenetic tree was constructed using the Bayesian algorithm MrBayes. Trees were edited and formatted using MEGA7.

### COI barcoding

To verify the species of our field-collected colony, part of the mitochondrial *cytochrome c oxidase* subunit I (COI) gene was sequenced and searched against the BLAST non-redundant nucleotide database. DNA was extracted from two clutches of eggs by pestling in 100 ul of DNA extraction buffer (10 mM Tris–Cl pH 8.2, 1 mM EDTA, 25 mM NaCl, and 200 μg/ml proteinase K), incubating at 37 °C for 30 min, then 95 °C for 2 min to inactive the proteinase K, then briefly spun down to pellet the yolk proteins and chorion. PCR was performed using 1 uL of the supernatant with the primers LCO1490 and HC02198 described in Folmer et al. [69] at an annealing temperature of 52 °C and an extension time of 15 s. The sequence of the PCR product was queried against NCBI’s non-redundant nucleotide database and the Barcode of Life Database (BOLD; [70]). The NCBI database search revealed matches to two *M. histrionica* specimens whose sequences have also been deposited in the BOLD database (specimens 1 and 2 in Additional file 7: Fig. S6 with 16/658 and 4/658 mismatches relative to our sequence, respectively). The sequence with highest percent identity match to our own in the BOLD database had just 2/568 mismatches (specimen 4 in Additional file 7: Fig. S6). The COI sequence has been deposited to GenBank with accession number MT238119.

## Supplementary information

**Additional file 1: Figure S1.** Sexing *Murgantia* A female (left) and a male (right) are shown. Males can be distinguished from females by the lateral lobes of the genital capsule which are externally visible (indicated by arrows).

**Additional file 2: Figure S2.** Removal of pseudoperculum. a) An intact egg. Pseudoperculum (or cap) is outlined and indicated by arrow. b) An embryo with removed cap (arrow indicates opening). c) A fully dissected embryo.

**Additional file 3: Table S1.** Primers used in this study.

**Additional file 4: Figure S3.** Phylogenetic trees. Accession numbers are listed next to each ortholog used. a) *Mh*-*En* was compared to other Engrailed and Invected orthologs. b) *Mh*-*E75A* was compared other E75A and EcR orthologs. c) *Mh*-*run* was compared to RunX ortholog family members: *lozenge*, *runxA*, and *runxB*. d) *Mh*-*odd* was compared to *odd*, *sob and bowl* orthologs. e) *Mh*-*eve* was compared to *eve, lab,* and *pb* orthologs. f) Both isoforms of *Mh*-*prd* were compared orthologs of *prd, gsb*, and *gsb*-*n*. g) *Mh*-*slp* was compared to other slp orthologs and fork head domain-containing genes *croc* and *FoxG*. Numbers at nodes represent posterior probability.

**Additional file 5: Figure S4.** Sequence alignment of Odd-skipped-related genes within Pentatomomorpha. A protein sequence alignment of odd-skipped and its paralogs, brother of odd with entrails limited (bowl), and sister of odd and bowl (sob). Motif A is outlined with a black box; Motif B with orange; Motif C with blue; and Motif D with yellow. Odd, sob and bowl all contain Motif C. Motif A and B are found in bowl only; and Motif D in sob only.

**Additional file 6: Figure S5.** Gene structure schematics. Structure schematic of the genes isolated are shown. Schematics were drawn to show regions isolated. a) The partial sequence of *Mh*-*en* isolated includes the homeodomain and the 3′ UTR. b) The partial sequence of *Mh*-*E75A* isolated includes the 5′ UTR, the DNA binding domain and the ligand binding domain. c) The partial sequence of *Mh*-*eve* isolated includes the homeodomain and the 3′UTR. d) The full sequence of *Mh*-*odd* was isolated, this includes its signature zinc fingers, the 3′ and 5′ UTR. f) Two isoforms of *Mh*-*prd* were isolated. These were designated the names *Mh*-*prd*-*A* and *Mh*-*prd*-*B*. *Mh*-*prd*-*B* contains an insertion of 25 amino acids in the Paired domain. f) The partial sequence of *Mh*-*slp* isolated includes the fork-head domain with no 3′ or 5′ UTR isolated. g) The partial *Mh*-*run* sequence isolated includes the runt domain with no 3′ or 5′ UTR isolated.

**Additional file 7: Figure S6.** Sequence alignment of 658 bp of the mitochondrial *cytochrome c oxidase* subunit I (COI) gene. The sequence generated for this study from our lab colony is sequence 3, indicated by a red box. All other sequences were retrieved from the BOLD database; BOLD sequence IDs are as follows: 1) CNCHA926-11.COI-5P; 2) CNCHA1208-11.COI-5P; 4) BBHMA706-12.COI-5P; 5) BBHMA577-12.COI-5P; 6) BBHMA702-12.COI-5P. The locations at which specimens were collected is shown in the top row. Nucleotides which differ from the consensus sequence are highlighted.

## Data Availability

All data generated or analyzed during this study are included in this published article and its additional files; sequences will be deposited in GenBank upon acceptance of the manuscript for publication.
