# Supplementary figures and images for: Oncopeltus-like gene expression patterns in Murgantia histrionica, a new hemipteran model system, suggest ancient regulatory network divergence
Source: EvoDevo. 2020 Apr 22;11:9. doi: 10.1186/s13227-020-00154-x (PMC7178596; doi:10.1186/s13227-020-00154-x)

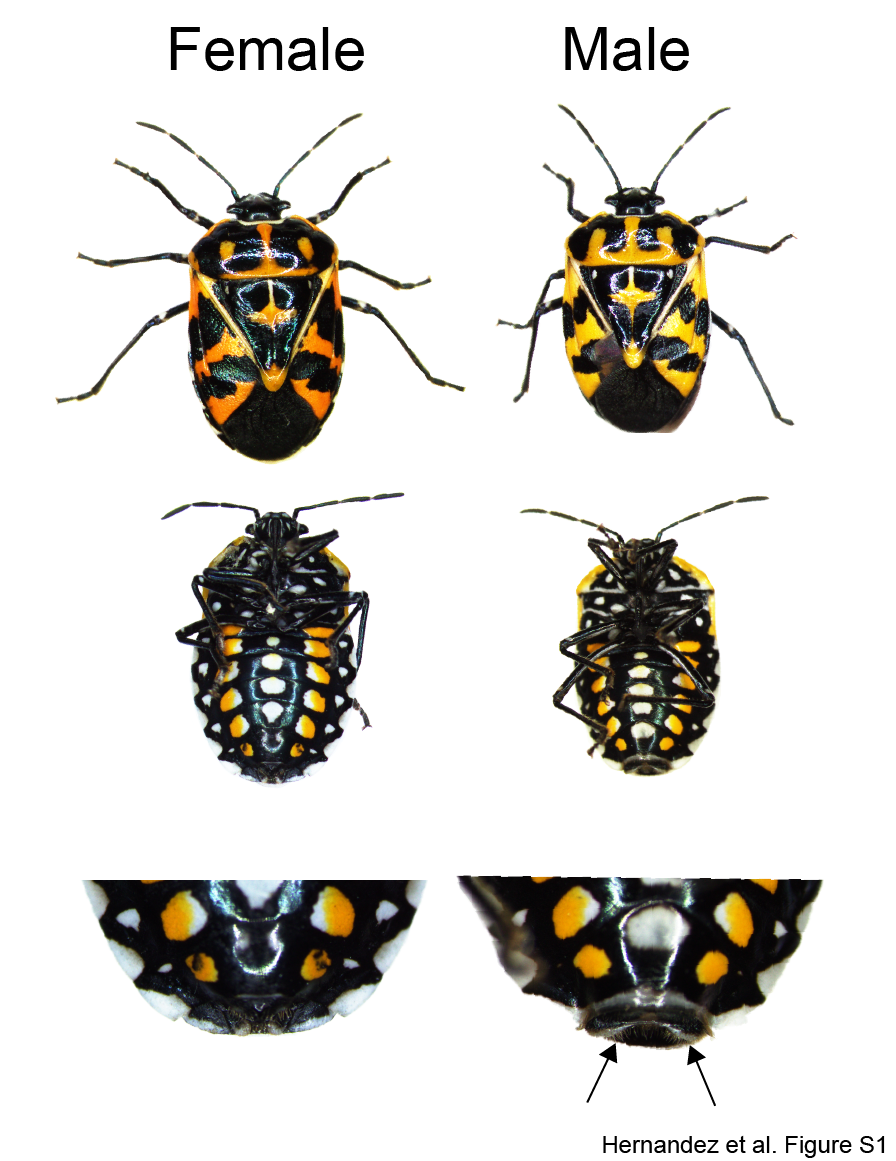

Supplement: Supplementary file 1 — Additional file 1: Figure S1. Sexing Murgantia A female (left) and a male (right) are shown. Males can be distinguished from females by the lateral lobes of the genital capsule which are externally visible (indicated by arrows). [file 13227_2020_154_MOESM1_ESM.png]

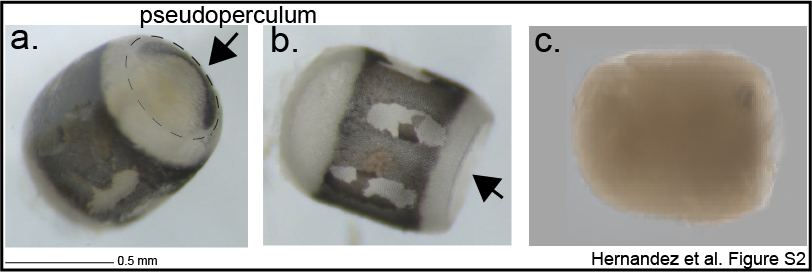

Supplement: Supplementary file 2 — Additional file 2: Figure S2. Removal of pseudoperculum. a) An intact egg. Pseudoperculum (or cap) is outlined and indicated by arrow. b) An embryo with removed cap (arrow indicates opening). c) A fully dissected embryo. [file 13227_2020_154_MOESM2_ESM.png]

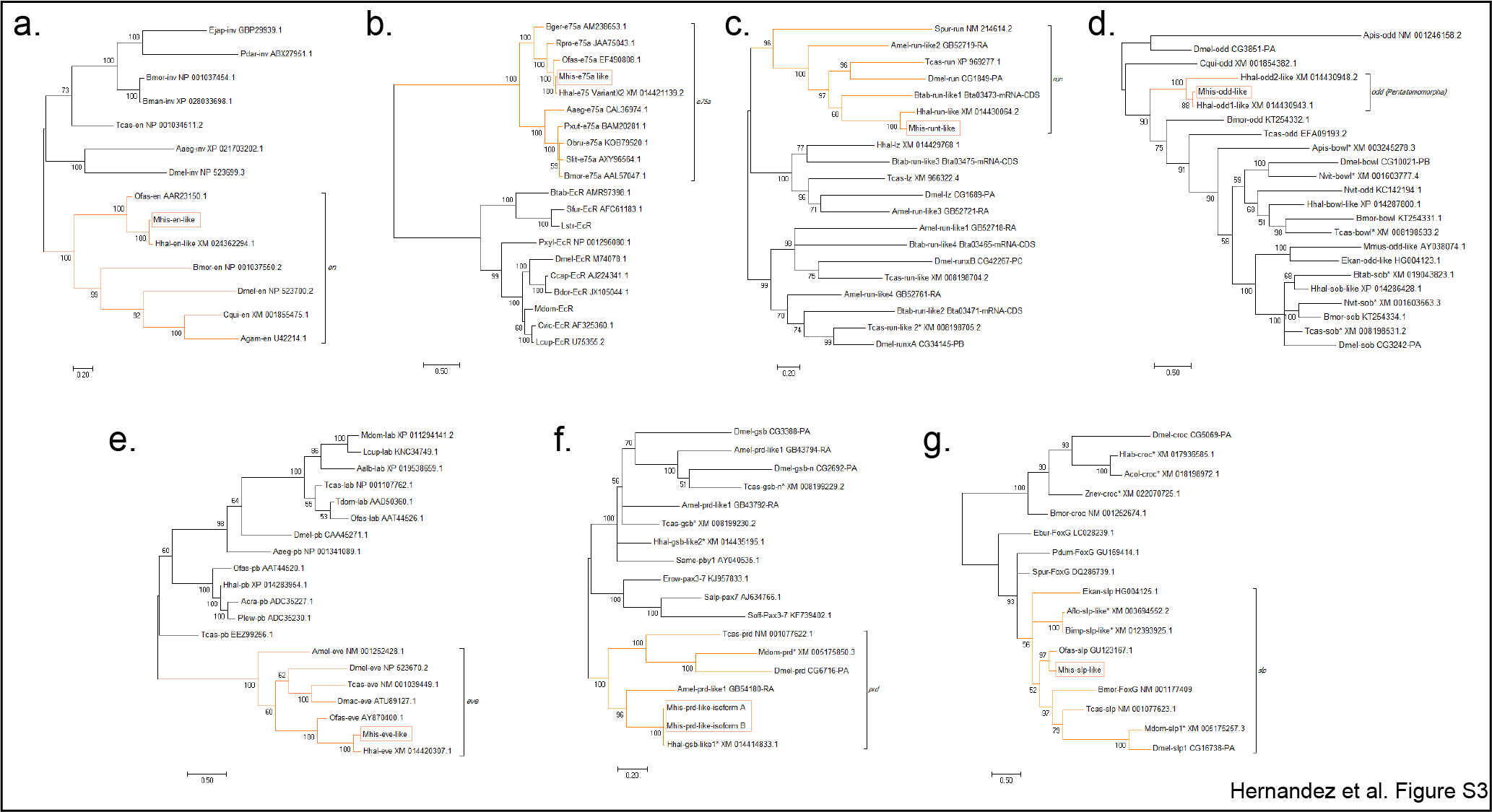

Supplement: Supplementary file 4 — Additional file 4: Figure S3. Phylogenetic trees. Accession numbers are listed next to each ortholog used. a) Mh-En was compared to other Engrailed and Invected orthologs. b) Mh-E75A was compared other E75A and EcR orthologs. c) Mh-run was compared to RunX ortholog family members: lozenge, runxA, and runxB. d) Mh-odd was compared to odd, sob and bowl orthologs. e) Mh-eve was compared to eve, lab, and pb orthologs. f) Both isoforms of Mh-prd were compared orthologs of prd, gsb, and gsb-n. g) Mh-slp was compared to other slp orthologs and fork head domain-containing genes croc and FoxG. Numbers at nodes represent posterior probability. [file 13227_2020_154_MOESM4_ESM.png]

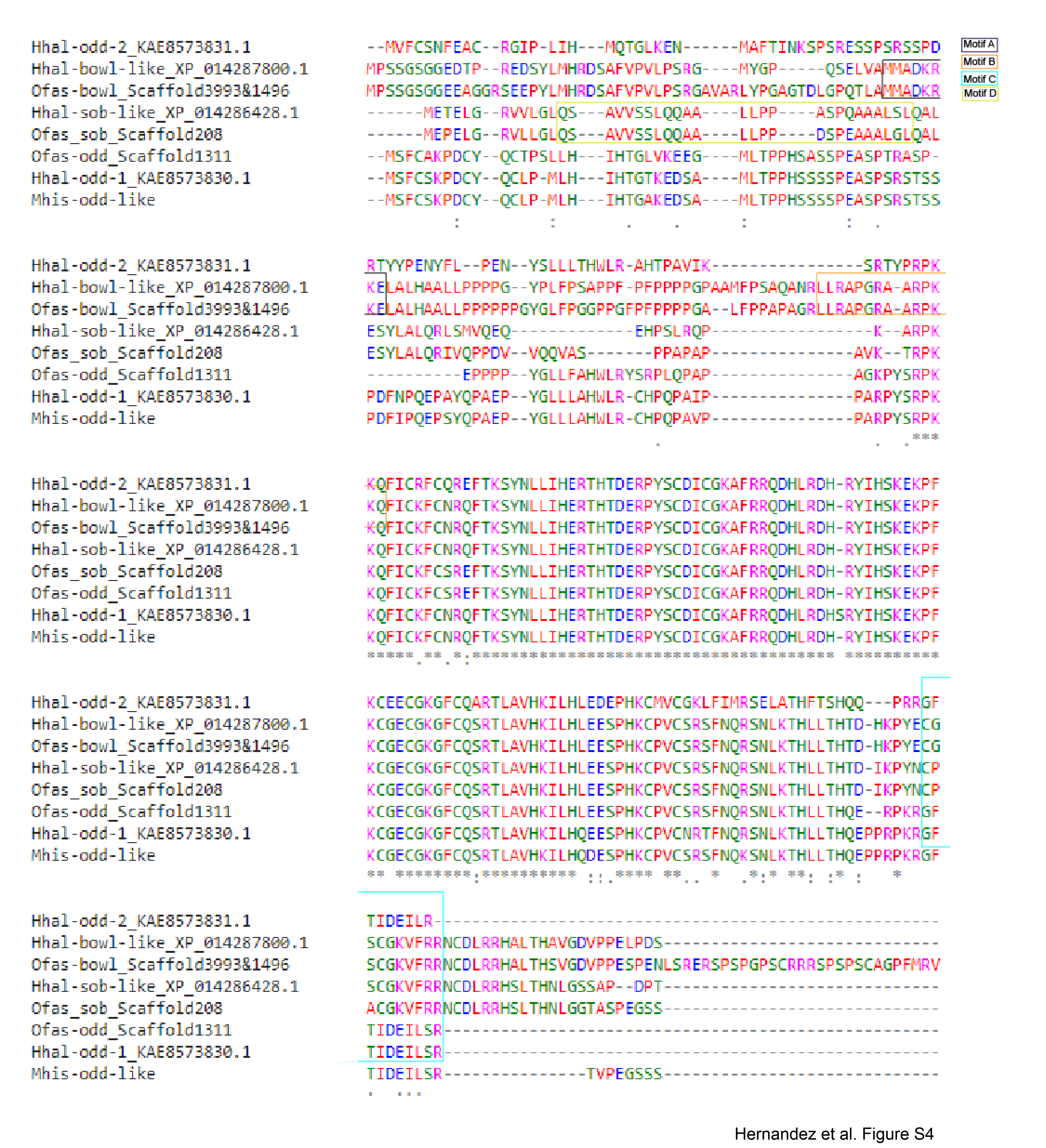

Supplement: Supplementary file 5 — Additional file 5: Figure S4. Sequence alignment of Odd-skipped-related genes within Pentatomomorpha. A protein sequence alignment of odd-skipped and its paralogs, brother of odd with entrails limited (bowl), and sister of odd and bowl (sob). Motif A is outlined with a black box; Motif B with orange; Motif C with blue; and Motif D with yellow. Odd, sob and bowl all contain Motif C. Motif A and B are found in bowl only; and Motif D in sob only. [file 13227_2020_154_MOESM5_ESM.png]

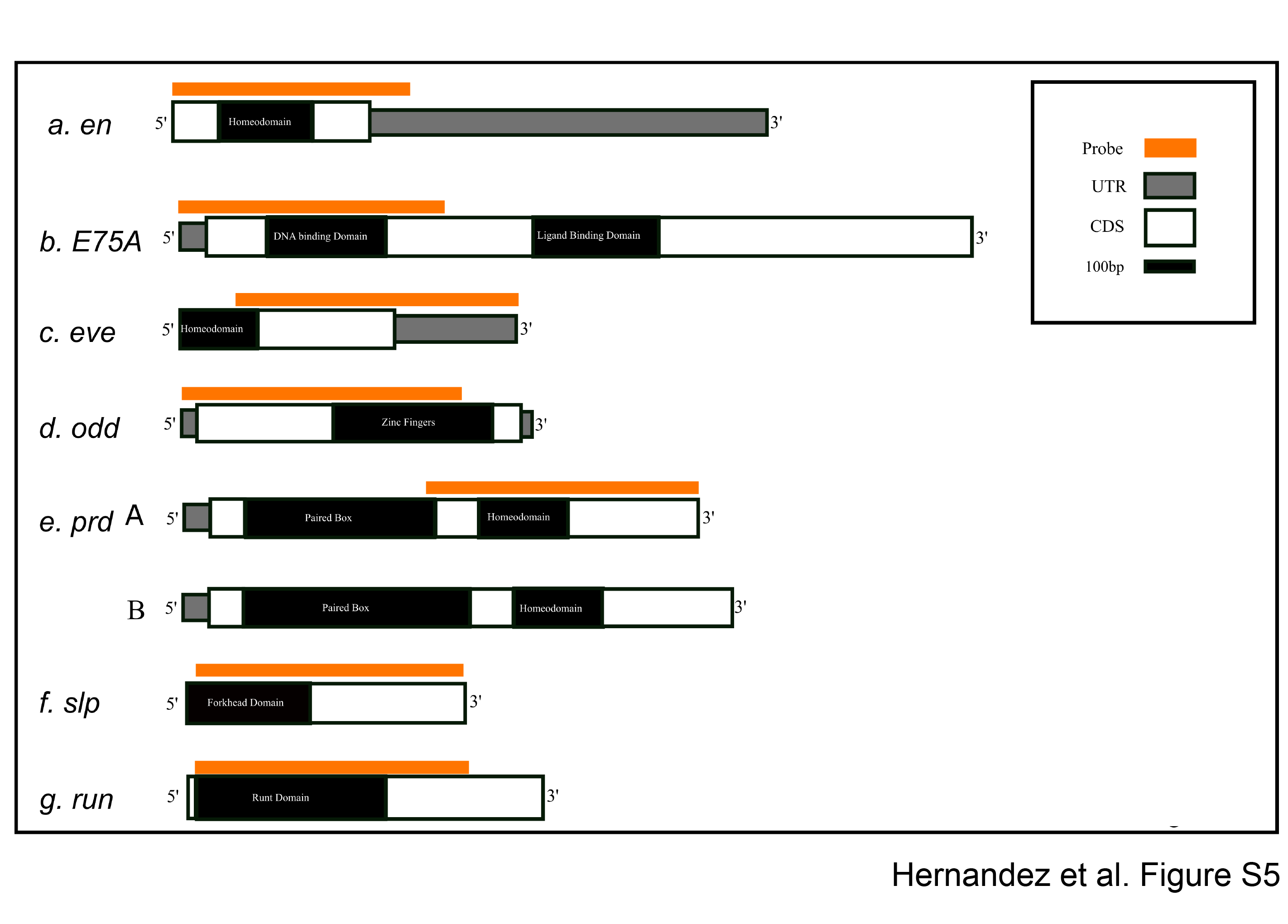

Supplement: Supplementary file 6 — Additional file 6: Figure S5. Gene structure schematics. Structure schematic of the genes isolated are shown. Schematics were drawn to show regions isolated. a) The partial sequence of Mh-en isolated includes the homeodomain and the 3′ UTR. b) The partial sequence of Mh-E75A isolated includes the 5′ UTR, the DNA binding domain and the ligand binding domain. c) The partial sequence of Mh-eve isolated includes the homeodomain and the 3′UTR. d) The full sequence of Mh-odd was isolated, this includes its signature zinc fingers, the 3′ and 5′ UTR. f) Two isoforms of Mh-prd were isolated. These were designated the names Mh-prd-A and Mh-prd-B. Mh-prd-B contains an insertion of 25 amino acids in the Paired domain. f) The partial sequence of Mh-slp isolated includes the fork-head domain with no 3′ or 5′ UTR isolated. g) The partial Mh-run sequence isolated includes the runt domain with no 3′ or 5′ UTR isolated. [file 13227_2020_154_MOESM6_ESM.png]

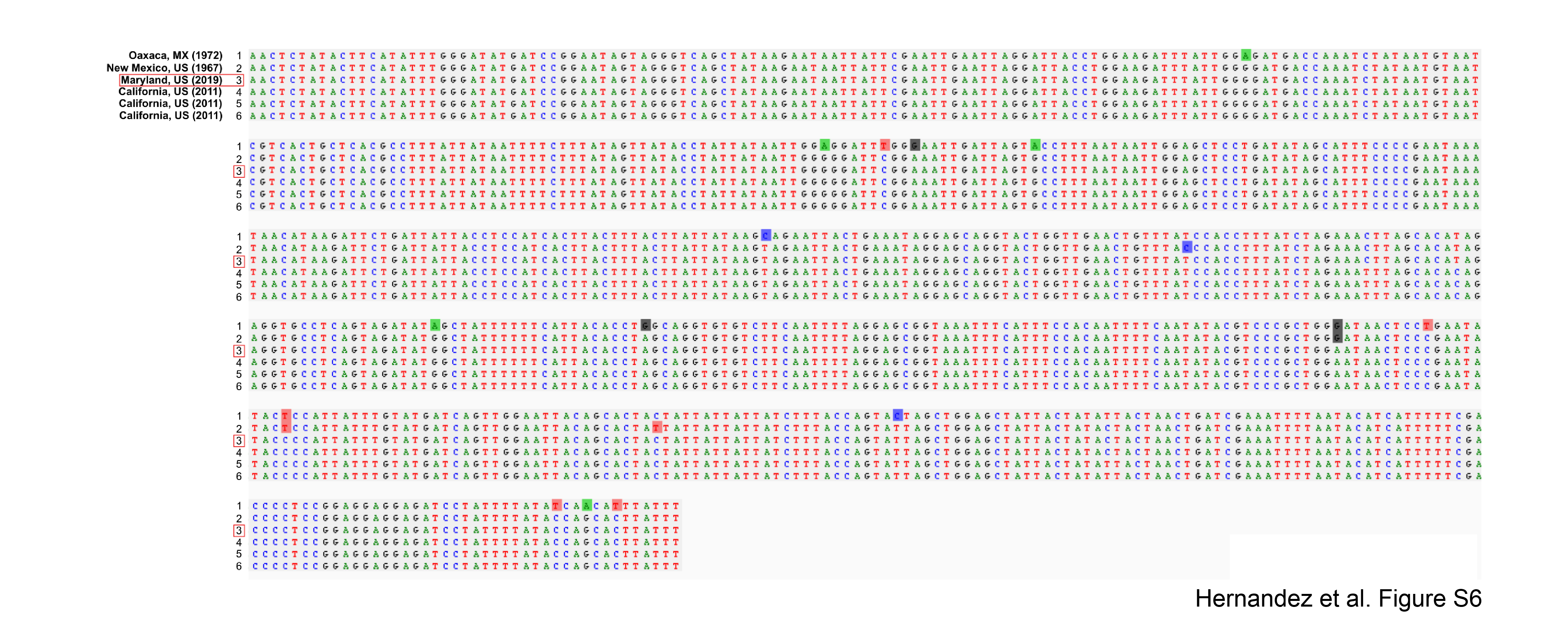

Supplement: Supplementary file 7 — Additional file 7: Figure S6. Sequence alignment of 658 bp of the mitochondrial cytochrome c oxidase subunit I (COI) gene. The sequence generated for this study from our lab colony is sequence 3, indicated by a red box. All other sequences were retrieved from the BOLD database; BOLD sequence IDs are as follows: 1) CNCHA926-11.COI-5P; 2) CNCHA1208-11.COI-5P; 4) BBHMA706-12.COI-5P; 5) BBHMA577-12.COI-5P; 6) BBHMA702-12.COI-5P. The locations at which specimens were collected is shown in the top row. Nucleotides which differ from the consensus sequence are highlighted. [file 13227_2020_154_MOESM7_ESM.png]
