# Supplementary material for: Oncopeltus-like gene expression patterns in Murgantia histrionica, a new hemipteran model system, suggest ancient regulatory network divergence
Source: EvoDevo. 2020 Apr 22;11:9. doi: 10.1186/s13227-020-00154-x (PMC7178596; doi:10.1186/s13227-020-00154-x)
Supplement: Supplementary file 3 — Additional file 3: Table S1. Primers used in this study. [file 13227_2020_154_MOESM3_ESM.docx]

**Additional Methods**

***engrailed* isolation:** *Mh-en* was isolated by degenerate PCR, using en-fw1 and en-bw4 (Damen, 2002) by touchdown PCR program with an annealing temperature starting at 50°C and decreasing by 0.5°C per cycle for 20 cycles, followed by an additional 20 cycles annealing at 40°C. The product was sequenced and searches of NCBI’s non-redundant protein database using the putative amino acid sequence as query and BLASTp returned a match to *Hhal-*En with 85.76% shared identity. The degenerate product sequence was used to design 3’ RACE primers Mhis-en-3’RACE-outer and Mhis-en-3’RACE-inner, and 3’ RACE was performed using the FirstChoice RLM-RACE Kit (Invitrogen). A product of about 1000 bp was obtained from the 3’RACE inner reaction. Mhis-en-3’RACE-outer and Mhis-en-R-1 were used to amplify a final partial product of 1167 bp using Phusion High-Fidelity DNA Polymerase (New England Biolabs). Finally, primers Mhis-en-3’RACE-outer and Mhis-en-R2-T7 were used to amplify 461 bp of *Mh-en* and used as probe template. In our phylogenetic analysis, Mh-En formed a clade with other En sequences suggesting that the product isolated was indeed *Mh-en* (Fig. S4a). The final *Mh-en* sequence isolated contains the partial coding sequence of *en* including the complete homeodomain sequence and the 3’ UTR (Fig. S3a).

***even-skipped* isolation:** Degenerate primers eve-fw, eve-bw-1 and eve-bw-2 (70) were used to isolate *Mh-eve*. Primers were used in a series of nested PCR reactions. The touchdown program described for *Mh-en* was used with eve-fw and eve-bw1 for an outer reaction. eve-fw and eve-bw2 were then used for an inner reaction using a standard Taq polymerase program with an annealing temperature of 50°C. A product of 138 bp was obtained from the inner reaction. BLASTp searches of NCBI’s non-redundant protein database showed that the *Mhis* sequence shared 91.30% identity with *Dmel-eve* and 93.48% identity with *Ofas-eve*. 3’ RACE primers were designed and used with the FirstChoice RLM-RACE Kit (Invitrogen). Mhis-eve-3’RACE-1 was used for an outer reaction and Mhis-eve-3’RACE-2 was used for an inner reaction. A product of 658 bp was obtained. Mhis-eve-3’RACE-1 and Mhis-eve-R2-T7 were used to amplify a final *Mh-eve* product of 698 bp. Finally, primers Mhis-eve-F-1 and Mhis-eve-R2-T7 were used to amplify 566 bp of *Mh-eve* and used as a template for a probe. In our phylogenetic tree of insect Eve proteins, Mh-Eve formed a clade with other Eve sequences suggesting that the product isolated was indeed Eve. The partial *Mh-eve* sequence isolated contains the homeodomain and the 3’ UTR (Fig S3c).

***E75A* isolation:** *Mh-E75A* was isolated by degenerate nested PCR. Primers DegenE75f and DegenE75r were used for an outer reaction using touchdown PCR. An inner reaction was performed using primers DegenE75f2 and DegenE75r1 with an annealing temperature of 43º C, yielding a product of 1138 bp. Mhis-E75-5’RACE-outer and Mhis-E75-5’RACE-inner were used in a series of nested PCR reactions. Multiple products were obtained from the 5’ RACE inner reaction. However, only the product containing both zinc fingers in the DNA binding domain (isoform A) was used in further experiments. A final product of 1558 bp was obtained using the primers *Mhis*-E75A-F1 and *Mhis*-E75A-R-4*.* Finally, 517 bp of *Mh-E75A* were amplified using *Mhis*-E75A-F1 and *Mhis*-E75A-R1-T7 and used as a template for probe synthesis. In our gene tree, *Mh*-E75A formed a clade with *Ofas*-E75A and *Hhal*-E75A suggesting that the product was indeed E75A. We isolated the partial sequence of *Mh-E75A* including the DNA binding domain, ligand binding domain and 5’UTR (Fig. S3b).

***runt* isolation**: *Mh-run* was isolated using a set of *Halyomorpha halys* (*Hhal)* primers: Hh-run-F3 and Hh-run-R1. A product of 760 bp was obtained; the sequence was trimmed to exclude the Hhal primer sequences and a final *Mh-run* product of 720 bp was used for further experiments. BLASTp revealed that the *Mhis* sequence shares 92.2% identity with *Hhal-run*. Primers Mhis-run-F1 and Mhis-run-R1-T7 were used to amplify 548 bp of *Mh-run* and used as a template for probe synthesis. A phylogenetic analysis was conducted using protein sequences of *run* along with the other Runt domain family members: *lozenge*, *runxA*, and *runxB* from various insect species (Fig. S4f). *Mh*-Run formed a clade with other Run orthologs including *Dmel*-Run suggesting that the product isolated was indeed *run*. The final *Mh-run* sequence contains the signature Runt domain (Fig. S3g).

***sloppy paired* isolation:** *Mh-slp* was isolated using the *Hhal* primers: Hh-slp-F2 and Hh-slp-R1. A standard Taq program with an annealing temperature of 50°C was used and 608 bp were isolated and sequenced. The nucleotide sequence was trimmed to exclude *Hhal* primer sequences and only 563 bp of *Mh-slp* were used for further experiments. BLASTp showed that this sequence shares 92.1% identity with *Hhal-slp*. Finally, primers Mhis-slp-F1 and Mhis-slp-R1-T7 were used to amplify 535 bp of *Mh-slp* as a probe template. Phylogenetic analysis showed that the product isolated is likely *slp* as it formed a clade with other Slp orthologs including *Ofas-*Slp *, Tcas-*Slp and *Dmel-*Slp (Fig. S3f; Fig. S4e).

***odd-skipped* isolation:** *Mh-odd* was isolated using *Hhal* primers: Hh-odd1-F-2 and Hh-odd1-R-1. A product of 531 bp was obtained using standard Taq program with an annealing temperature of 50°C. RACE primers Mhis-odd1-5’RACE-R-1 and Mhis-odd1-3’RACE-F-1 were designed and used with the SMARTer RACE 5’/3’ Kit (Takara). 5’RACE yielded a product of 221 bp while the 3’RACE produced a product of 601 bp. Primers Mhis-odd-F-1 and Mhis-odd-R-1 were used to amplify a final *Mh-odd* product of 712 bp. Finally, primers Mh-odd-F-1 and Mhis-odd-R2-T7 were used to amplify 560 bp of *Mh-odd* which was used as a template for probe synthesis. *odd* has two paralogs: *brother of odd with entrails limited (bowl)* and *sister of odd and bowl (sob)*. Thus protein sequences of *odd, bowl*, and *sob* from various insects were compared through phylogenetic analysis (Fig. S4g). *Mh*-Odd formed a clade with *Ofas-*Odd and *Hhal-*Odd suggesting that the product isolated was indeed *odd*. Further, protein alignment confirmed that the sequence isolated lacks motifs A, B and D, those found in *sob* and *bowl* (21). The final sequence isolated includes the complete coding sequence of *Mh-odd* containing its signature zinc fingers (Fig. S6).

***paired* isolation:** Degenerate PCR was used to isolate *Mh-prd* in a series of nested reactions. The outer reaction was performed with primers prd-fw3 and prd-bw1 using touchdown PCR. An inner reaction was performed with primers prd-fw1 and prd-bw3 using a standard Taq program with an annealing temperature of 45°C. The inner reaction produced three products: two isoforms of *Mh-prd* as well as *Mh-gooseberry (gsb)*. One of the isoforms of *Mh-prd* contained an insertion of 75 bp (25 amino acids) within the Prd domain in the sequence WEIRD-RLIKE (hyphen indicates location of insertion). RACE primers were designed and used with the SMARTer RACE 5’/3’ Kit (Takara). Primers were: Mhis-prd-3’RACE-F1 and Mhis-prd-5’RACE-R1. Primers Mhis-prd-F-1and Mhis-prd-R1-T7 were used to amplify two final *Mh-prd* products of 1043 bp and 1108bp. Finally, primers Mhis-prd-F-2 and Mhis-prd-R1-T7 were used to amplify 545 bp of *Mh-prd* and used as a template for probe synthesis. Both sequences were subjected to phylogenetic analysis and compared to protein sequences of *prd*, *gooseberry* (*gsb)*, and *gooseberry-neuro* (*gsb-n*) from several insect species (Fig. S4d). The sequence isolated includes the paired box and the homeobox.

**Table S1.** Primers used in this study

| Name | Sequence |
| --- | --- |
|  |  |
| en-fw1 | TGGCCMGCMTGGGTNTWYTGYAC |
| en-bw4 | TTRTAMARNCCYTSNGCCAT |
| en-bw3 | RTTYTGRAACCADATYTTDATYTG |
| Mhis-en-3’RACE-outer | CAGCCTGGGTTTATTGTACCAGA |
| Mhis-en-3’RACE-inner | CAGATCAAGATCTGGTTCCAGAACA |
| Mhis-en-5’RACE-outer | TCTTGTTCTGGAACCAGATCTTGAT |
| Mhis-en-5’RACE-inner | CTGAAGGCAGTCCTTGGTCT |
| Mhis-en-R-1 | AATAGGGATAGCATAATAGACGTCACAATAAT |
| Mhis-en-R2-T7 | CTGAGACCTATCTGAGGACG |
| eve-fw | ACNGCNTTYACNMGNGARCA |
| eve-bw-1 | CKYTGNCKYTTRTCYTTCAT |
| eve-bw-2 | RTTYTGRAACCANACYTTDATNGT |
| Mhis-eve-3’RACE-1 | ACAAGTGAGCAACTGAGCAGG |
| Mhis-eve-3’RACE-2 | AGTCTACCATCAAGGTATGGTTCC |
| Mhis-eve-R2-T7 | TGATGGGGTTCAGCAATATTTTATTTGTAAT |
| Mhis-eve-F-1 | AGGATGAAAGACAAGCGGCA |
| DegenE75f1 | GAYAARGCNGGNTTYCAYTA |
| DegenE75f2 | ATHCARCARAARATHCARTA |
| DegenE75r1 | AAYTTYTTRTGNGGYTTRTA |
| Mhis-E75-5’RACE-outer | CTGGAGTCGTCTTCCAGTTCT |
| Mhis-E75-5’RACE-inner | GGTACTCTGCTGCATTGCTG |
| Mhis-E75a-5’RACE-inner | GCATCCTTCGCAGGAGTGTA |
| Mhis-E75A-F-1 | ATTGACAGTAGAGTGCCGAAGT |
| Mhis-E75A-R-4 | TTCTCAGTCCCCGATTCGATC |
| Mhis-E75A-R1-T7 | CTGGAGTCGTCTTCCAGTTCT |
| Hh-run-F3 | GTGGAACGGGAGAACTCTGG |
| Hh-run-R1 | GTGGAACGGGAGAACTCTGG |
| Mhis-run-F1 | TCCCAACCACTGGAGGTCTAATAA |
| Mhis-run-R1-T7 | GCGGCAGTAGTTCGCTAC |
| Hh-slp-F2 | AAACCACCATACAGTTACAACGCC |
| Hh-slp-R1 | GTGTGAGTAGCCAAATGTGTC |
| Mhis-slp-F1 | ATGATGGCGATCCGACAGAGT |
| Mhis-slp-R1-T7 | CGATCACAGTCACAGGCTTGAAG |
| Hh-odd1-F-2 | ATGTCGTTTTGCTCTAAACCAGATTG |
| Hh-odd1-R-1 | CTTCAGGTTGGATCGTTGGTTG |
| Mhis-odd1-3'RACE-F-1 | AACCTTTCAAATGCGGTGAGTGCGGAAAAGGCT |
| Mhis-odd1-5’RACE-R-1 | GCTGGTTGGTAGGATGGCTCCTGAGGGATG |
| Mhis-odd-F-1 | GGGACAGTCAGAACAGATAGCA |
| Mhis-odd-R-1 | TGAACTTGAACCCTCGGGTAC |
| Mhis-odd-R2-T7 | GAGATTCGTCCTGGTGCAAGA |
| prd-fw1 | GGNGGNGTNTTYATHAAYGG |
| prd-fw3 | CARGGNMGNGTNAAYCAR |
| prd-bw1 | RTTNSWRAACCANACYTG |
| prd-bw3 | YTCYTCNCKNGTRTADATRTC |
| Mhis-prd-3’RACE | GAAGGACTCTGCGACCGAGGTAACGCCC |
| Mhis-prd-5’RACE | GGGCGTTACCTCGGTCGCAGAGTCCTTCCT |
| Mhis-prd-F-1 | TAGACACCACCAGAGACAGGAG |
| Mhis-prd-R1-T7 | GGTAGTGAGAAGCGTTGTGGATG |
| Mhis-prd-F-2 | TACGGGGAAACGACGGAGAA |
| Mhis-prd-R1-T7 | GGTAGTGAGAAGCGTTGTGGATG |
